# TSH−SPP1/TRβ−TSH positive feedback loop mediates fat deposition of hepatocyte: Crosstalk between thyroid and liver

**DOI:** 10.3389/fimmu.2022.1009912

**Published:** 2022-10-10

**Authors:** Bin Huang, Wenjie Wen, Shandong Ye

**Affiliations:** ^1^ Department of Endocrinology, The First Affiliated Hospital of University of Science and Technology of China (USTC), Division of Life Science and Medicine, University of Science and Technology of China, Hefei, China; ^2^ Division of Life Sciences, University of Science and Technology of China, Hefei, China

**Keywords:** non-alcoholic fatty liver disease, obesity, thyroid function, TSH, thyroid hormone receptor, SPP1, M1 macrophage polarization, positive feedback crosstalk

## Abstract

**Aims:**

We conducted this study with two aims: (1) whether TRβ could be damaged by NAFLD, thereby represent thyroid hormone resistance-like manifestation and (2) to analyze the potential role of SPP1 in TH signaling pathway on the process of NAFLD. This study is expected to provide a new perspective on the therapeutic mechanism in the pathological course of NAFLD.

**Methods:**

A total of 166 patients diagnosed with type 2 diabetes mellitus (T2DM) were enrolled in this study. All patients had a BMI above 24 kg/m2 and were stratified into two groups: NAFLD and Non-NAFLD groups. Ages, gender, BMI, duration of diabetes and biochemical markers were obtained from participants’ records. We downloaded the dataset GSE48452 from GEO. The Pathview library was used to make the thyroid hormone signaling pathway visualization. The CIBERSORT algorithm was applied to calculate the infiltrated immune cells in obese NAFLD patients. C57BL/6 mice were randomly selected to constitute the normal control (NC) group and were fed a normal chow diet; the rest of the mice were fed a high-fat diet (HFD). After 12 weeks HFD feeding, the mice were sacrificed by cervical dislocation, and blood samples were collected. Mouse livers were also collected; one part of each liver was fixed in 10% formalin for histological analysis, and the other part was snap-frozen for subsequent molecular analyses. To explore the relationship between SPP1, TRβ and lipid deposition in hepatocytes, HepG2 cells were treated with 50 μ M concentration of PA and/or 20 ng/ml concentration of rh-SPP1 for 48h. In addition, the PC3.1-TRβ plasmid was constructed for further validation in HepG2 cells. We used THP-1 cells to construct an M1 macrophage model *in vitro*. We then analyzed THP-1 cells treated with various concentrations of PA or TSH.

**Results:**

(1) After adjusting for all factors that appeared P value less than 0.1 in the univariate analysis, BMI, TSH, and FT3 were significant independent risk factors of NAFLD (ORs were 1.218, 1.694, and 2.259, respectively); (2) A further analysis with BMI stratification indiacted that both FT3 and TSH had a significant change between individuals with NAFLD and Non-NAFLD in obesity subgroup; however, there was no statistic difference in over-weight group; (3) Bioinformatics analysis of GSE48452 had shown that several key molecular (including TRβ) of thyroid hormone pathway affected by NAFLD induced transcriptomic changes and the expression levels of SPP1, FABP4 and RPS4Y1 were significantly higher, while the expression levels of PZP and VIL1 were significantly decreased in NAFLD patients(adjusted p < 0.05, |logFC| > 1.0). The CIBERSORT algorithm showed increased M0 and M1, decreased M2 macrophage infiltration in NAFLD with comparison to healthy obese group; (4) After 12 weeks of HFD-feeding, the obesity mice had significantly higher serum TSH and In IHC-stained liver sections of obesity group, the intensity of SPP1 had a significantly increased, while TRβ reduced; (5) *In vitro* studies have shown SPP1 aggravated lipid deposition in hepatic cells dependent on down-regulating the expression of TRβ and TSH acts to promote secretion of SPP1 in M1 macrophage cells.

**Conclusions:**

SPP1 secretion induced by M1 macrophage polarization, which may down-regulates TRβ in hepatocytes *via* paracrine manner, on the one hand, the lipid deposition aggravating in liver, on the other hand, a compensatory increase of TSH in serum. The increased TSH can further lead to the following SPP1 secretion of M1 macrophage. The positive feedback crosstalk between thyroid and liver, may be plays an important role in maintaining and amplifying pathological process of NAFLD.

## Introduction

Non-alcoholic fatty liver disease (NAFLD), now known as metabolic dysfunction-associated fatty liver disease (MAFLD), affects approximately one-quarter of the world’s adult population and poses a major health and economic burden to all societies (1). The global obesity pandemic, which has grown over the past three decades, has been a major cause of this dramatic increase in the incidence of NAFLD (2). In general, NAFLD is considered to be the hepatic manifestation of metabolic syndrome, which is most commonly observed in cases of obesity and/or type 2 diabetes mellitus (T2DM), and not only constitutes a first stage in this disease, but the toxicity exerted by certain lipids might also drive further steps in this disease, such as inflammation and liver injury (3). The rise in NAFLD has led to a remarkable increase in the number of cases of cirrhosis, hepatocellular carcinoma, hepatic decompensation, and liver-related mortality related to NAFLD (4). It is currently believed that chronic inflammation caused by excessive deposition of liver fat and changes in the local immune environment are important mechanisms that lead to the transformation of NAFLD to hepatocellular carcinoma (HCC) (5, 6). Therefore, the identification of mechanisms in the process of liver lipid deposition may provide important targets for the prevention of NAFLD-associated HCC.

Thyroid hormone are known to have significant effects on lipid metabolism in that live (7, 8). Hypothyroidism-induced NAFLD is often attributed to disruption of thyroid hormone (TH) signaling, resulting in decreased hepatic utilization of lipids. In fact, subclinical hypothyroidism, even in the higher range of normal serum thyroid-stimulating hormone (TSH) concentrations, has been found to be dose-dependently associated with NAFLD (9, 10). In our previous study, 369 euthyroid T2DM individuals with suspected NAFLD were involved, higher levels of free triiodothyronine (FT3) and TSH were observed than in individuals without NAFLD, confirming the existence of a thyroid hormone resistance-like manifestation in these patients (11). There are two major isoforms of the thyroid hormone receptor (TR), TRα and TRβ, which are the predominant receptors in the liver (12). To date, however, there has been little research on the association between TRβ and NAFLD, which may involve the intrinsic mechanism of NAFLD development. Secreted phosphoprotein 1 (SPP1), also known as osteopontin, is expressed in a variety of tissues, including adipose, liver, and kidney, as well as macrophages. Studies have shown that in both animal models and human experiments, SPP1 can mediate chronic inflammation *in vivo* and promote fat deposition and malignant lesions in the liver (13, 14). However, studies on the role of SPP1 in the TH signaling pathway in the liver are inconclusive.

Accumulation of liver fat, which is most commonly observed in cases of obesity or T2DM, might drive further steps in diseases, such as inflammation, liver injury, and insulin resistance (15). Hence, we designed a study for individuals diagnosed with T2DM and being overweight/obese, making them suitable for the diagnosis of MAFLD, and to be adjusted for the interference from metabolic dysfunction beyond the liver. This study was conducted with two aims: (1) to determine whether TRβ could be damaged by NAFLD, thereby representing TH resistance-like manifestation and (2) to analyze the potential role of SPP1 in the TH signaling pathway in NAFLD. This study provides a new perspective on the therapeutic mechanisms underlying the pathological course of NAFLD.

## Materials and methods

### Study population

A total of 166 patients diagnosed with T2DM were enrolled in this study from the Department of Endocrinology of the First Affiliated Hospital (Anhui Provincial Hospital) of the University of Science and Technology of China (USTC) between July 2017 and September 2018. All patients had a body mass index (BMI) above 24 kg/m^2^ (considering ethnic differences, our study adopted the obesity diagnostic criteria recommended by the World Health Organization (WHO) for Chinese people: BMI ≥ 24 kg/m^2^ is overweight and BMI ≥ 28 kg/m^2^ is obesity) and normal thyroid function (euthyroid, defined as both free thyroxine and TSH within the reference range). The requirement for informed consent was waived because this study was designed to retrospectively collect available data from the participants’ medical records. Patients were stratified into two groups as mentioned previously: NAFLD and healthy liver groups. All people included in this study had T2DM, so they were in line with the diagnosis of MAFLD. However, to be consistent with our previous study, NAFLD was used in this study. Patients with acute complications from diabetes, severe hepatic disease (if the value of liver function index (alanine transaminase or aspartate transaminase) exceeded the upper normal reference value by 1.5×), severe chronic kidney disease (defined as estimated glomerular filtration rate ≤ 60 mL/min/1.73 m^2^), cancer, or other severe coexisting illnesses were excluded from the study.

### Clinical and laboratory evaluation

Age, sex, BMI, and duration of diabetes were obtained from the participants’ records. All patients were tested for biochemical markers of liver function: alanine transaminase (ALT) and aspartate transaminase (AST); kidney function: creatinine (Cr); glucose metabolism: fasting blood glucose (FBG), fasting insulin (Fins), fasting C-peptide (FCP), and hemoglobin A1c (HbA1c); lipid metabolism: triglycerides (TG), total cholesterol (TC), low density lipoprotein cholesterol (LDL-c), and high-density lipoprotein cholesterol (HDL-c); and thyroid function: FT3 (normal range: 3.28–6.47 pmol/L), free thyroxine (FT4, normal range: 7.90–19.05 pmol/L), and TSH (normal range: 0.350–4.949 mIU/L) at baseline.

### Bioinformatics analysis

The dataset GSE48452 was downloaded from the Gene Expression Omnibus (GEO; http://www.ncbi.nlm.nih.gov/geo). In the dataset, the samples were divided into two groups (24 obese patients with NAFLD and 16 with a healthy liver). After consolidation and normalization of the RNA-seq data, 118 differentially expressed genes (DEGs) involved in NAFLD were screened using the limma package (adjusted p < 0.05, |logFC| > 0.5). The Pathview library (https://pathview.uncc.edu/home) was used to visualize the TH signaling pathway. The Cell type Identification by Estimating Relative Subsets of known RNA Transcripts (CIBERSORT) algorithm was applied to calculate the infiltrated immune cells in obese patients with NAFLD.

### Animal experiments

Six-week-old specific pathogen-free C57BL/6 mice weighing 20–23 g were purchased from the Shanghai Model Organisms Center, Inc. (Shanghai, China). The animal protocols were approved by the Animal Care and Use Committee of University of Science and Technology of China (Approval ID: 2022-N(A)-043). All mice were maintained in a 48 ± 10% humid environment at room temperature (20 ± 1°C) under a 12-h light/dark cycle, with free access to food. Every effort was made to minimize the number of animals used and their suffering. Eight mice were randomly selected to constitute the normal control (NC) group and fed a normal chow diet, while the remaining mice were fed a high-fat diet (HFD). After 12 weeks, the mice were fasted overnight, anesthetized by intraperitoneal injection of sodium pentobarbital (30 mg/kg), euthanized by cervical dislocation, and then blood samples and livers were collected. One part of each liver was fixed in 10% formalin for histological analysis, and the remaining part was snap-frozen for subsequent molecular analyses.

### Enzyme-linked immunosorbent assay

Collected blood or cell supernatant samples were centrifuge at 3,000 rpm for 10 min. SPP1 levels were determined using a commercial ELISA kit (Meimian, Jiangsu, China) and optical density was measured at 450 nm. TSH levels were measured using the picrate method (Jiancheng, Nanjing, China) and optical density was measured at 450 nm. The kits were used in accordance with the manufacturer’s instructions.

### Immunohistochemistry staining

Liver tissues were fixed with 10% buffered formalin for 48 h and embedded in paraffin. A microtome was used to cut 4–6 μm sections for tissue analysis. Liver sections were deparaffinized, antigen was retrieved, endogenous catalase was removed with 3% H_2_O_2_, incubated in blocking solution, and then incubated with anti-SPP1 (1:200, Proteintech), TSH (1:200, Bioss), and TRβ (1:200, Bioss) antibodies overnight at 4°C. Sections were then incubated with HRP-conjugated secondary antibodies and developed with 3,3-diaminobenzidine.

### Cell culture and treatment

HepG2 cells were maintained in Dulbecco’s modified Eagle medium supplemented with 25 mmol/L glucose, 10% fetal bovine serum (FBS), 100 U/mL penicillin, and 100 mg/mL streptomycin and were cultured at 37 °C in a 95% humidity and 5% CO2-containing environment. Bovine serum albumin-conjugated palmitic acid (PA; Sigma-Aldrich, St. Louis, MO, USA) was prepared as previously described (16). To explore the relationship between SPP1, TRβ and lipid deposition in hepatocytes, HepG2 cells were treated with 50 μ M concentration of PA and/or 20 ng/ml concentration of rh-SPP1 (MedChemExpress,China) for 48h. In addition, the PC3.1-TRβ plasmid was constructed for further validation in HepG2 cells. Human leukemia monocyte THP-1 cells were maintained in RPMI 1640 medium containing 10% FBS, 100 U/mL penicillin, and 100 mg/mL streptomycin and were cultured at 37 °C in a 95% humidity and 5% CO2-containing environment. To obtain THP-1-derived macrophages, THP-1 cells were treated as previously described (17). Briefly, THP-1 macrophages were treated with 500 ng/mL phorbol 12-myristate 13-acetic acid (PMA) for 72 h to induce THP-1 differentiation into macrophages (THP-1 M0 macrophages), followed by induction of M1 macrophage activation by 200 ng/mL lipopolysaccharide. To verify whether TSH promotes the secretion of SPP1 in M1 macrophages. We used THP-1 cells to construct an M1 macrophage model *in vitro*. We then analyzed THP-1 cells treated with various concentrations of PA or TSH.

### Oil red O staining

HepG2 cells were seeded in 12-well plates, washed three times with phosphate buffered saline (PBS), and fixed with 4% formaldehyde for 30 min. Oil Red O (0.5% in isopropanol), filtered through a 0.45 mm filter, and then added to the fixed cells for 20 min at room temperature. Cells were washed with 70% ethanol and water, observed, and photographed using a light microscope. Mouse livers were fixed with 4% paraformaldehyde (PFA) at 4°C for 24 h. Subsequently, the precipitate was dehydrated with 20 and 30% sucrose solutions. The surface water was gently absorbed with filter paper, liver samples were embedded with optimal cutting temperature (OCT), frozen, and sectioned after the OCT complex became white and hard. It was then soaked in 85 or 100% propylene glycol for 6 min. Next, the sections were incubated for 2 h at room temperature with freshly prepared 0.5% Oil Red O dye solution. The background color was removed using 100% propylene glycol for 30 s. The dye solution was gently rinsed with PBS. The sections were re-stained with hematoxylin dye solution for 10 s, rinsed with water, and sealed with gelatin. Finally, lipid droplets (stained red) were observed under a microscope and imaged.

### Western blotting

For western blotting, total proteins in liver tissues and cells were extracted by grinding the tissues in radioimmunoprecipitation assay buffer. Protein concentration was detected using a BCA kit and then adjusted to equal levels in all samples. Protein loading buffer was added (total protein:loading buffer = 4:1) and samples were heated at 95°C for 5 min, then cooled on ice. Samples were then separated on 10% sodium dodecyl sulfate-polyacrylamide gel electrophoresis gels and then transferred to nitrocellulose filter membranes. The membranes were blocked with 5% skim milk. Proteins were detected using specific primary anti-β-actin (1:5000, Enogene, Nanjing, China), anti-CD86 (1:1000, Bioss, Beijing, China), anti-TRβ (1:1000, Affinity, Jiangsu, China), anti-TSHR (1:1000, Affinity, Jiangsu, China), and anti-SPP1 (1:1000, Proteintech, Wuhan, China) antibodies. Protein bands were visualized using a Super ECL kit (UElandy, Suzhou, China) and analyzed using ImageJ software.

### Statistical analyses

The IBM SPSS Statistics ver. 24.0 (IBM). Continuous measurements, such as the mean and standard deviation (SD), were used if the data were normally distributed; however, if the data were not normally distributed, the median inter-quartile range (IQR) was used. Categorical variables were described as frequencies and percentages (%). Independent tests, including the t-test, Chi-squared test, or Mann–Whitney U test, were used to compare the two patient groups. Logistic regression analysis was used to calculate odds ratios (ORs) and 95% confidence intervals (CIs) for the risk of NAFLD, while adjusting for potential confounding variables. Spearman’s correlation analysis was used to describe the relationship between thyroid index and BMI. Statistical significance was set at p < 0.05.

## Results

### Demographic and metabolic characteristics of study subjects

The data of 166 patients with T2DM (71.6% male and 51.2% NAFLD) were evaluated. The mean age of the study subjects was 55.10 ± 12.89 years, ranging from 19–84 years. The duration of diabetes ranged from 0 to 35 years. Patients in the NAFLD group had significantly higher BMI and TSH levels than those in the non-NAFLD group (all p < 0.05). There were no significant differences in sex, age, duration of diabetes, FBG, HbA1c, Fins, FCP, TG, TC, LDL-c, HDL-c, ALT, AST, Cr, FT3, or FT4 between the two groups (all p > 0.05) (**Table 1**).

**Table 1:** Demographic and metabolism characterization of study subjects.

Characteristic	Non-NAFLD	NAFLD	p
n	81	85	
Gender, n (%)			0.402
Male	61 (36.7%)	58 (34.9%)	
Female	20 (12%)	27 (16.3%)	
Age (year)	55.98 ± 12.32	54.26 ± 13.66	0.397
BMI (Kg/m2)	26 (25.04, 27.68)	27.73 (25.78, 30.53)	< 0.001
Duration of diabetes (year)	7 (3, 12)	7 (1, 12)	0.504
FPG (mmol/L)	7.53 (6.38, 10.11)	8.4 (6.77, 10.16)	0.410
Fins (pmol/L)	65.63 (47.7, 80.47)	73.48 (47.67, 128.95)	0.358
F-CP (nmol/L)	0.42 (0.25, 0.62)	0.5 (0.36, 0.73)	0.142
HbA1C (%)	7.7 (6.6, 9.1)	8.5 (7.3, 10)	0.062
TG (mmol/L)	1.92 (1.43, 3.08)	1.97 (1.48, 3.02)	0.888
TC (mmol/L)	4.55 (3.95, 5.21)	4.78 (3.82, 5.3)	0.803
LDL (mmol/L)	2.51 ± 0.83	2.42 ± 0.78	0.475
HDL (mmol/L)	0.99 (0.84, 1.08)	0.96 (0.83, 1.05)	0.584
ALT (IU/L)	23 (17.25, 34)	24 (16, 45)	0.582
AST (IU/L)	22 (18, 27.75)	21 (16.75, 34)	0.700
Cr (mmol/L)	83 (73, 93)	80.5 (69.75, 89.5)	0.476
FT3 (pmmol/L)	5 (4.71, 5.35)	5.21 (4.78, 5.51)	0.071
FT4 (pmmol/L)	12.46 (11.38, 14.05)	12.58 (11.24, 13.69)	0.369
TSH (mIU/L)	1.78 (1.02, 2.57)	1.94 (1.58, 2.84)	0.034
25-OHD (ng/ml)	19.23 ± 7.06	18.41 ± 6.91	0.502

Data are shown as n (%) and as either mean ± SD or median (interquartile range, IQR).

### Independent risk factors associated with the incidence of NAFLD

**Figure 1:**
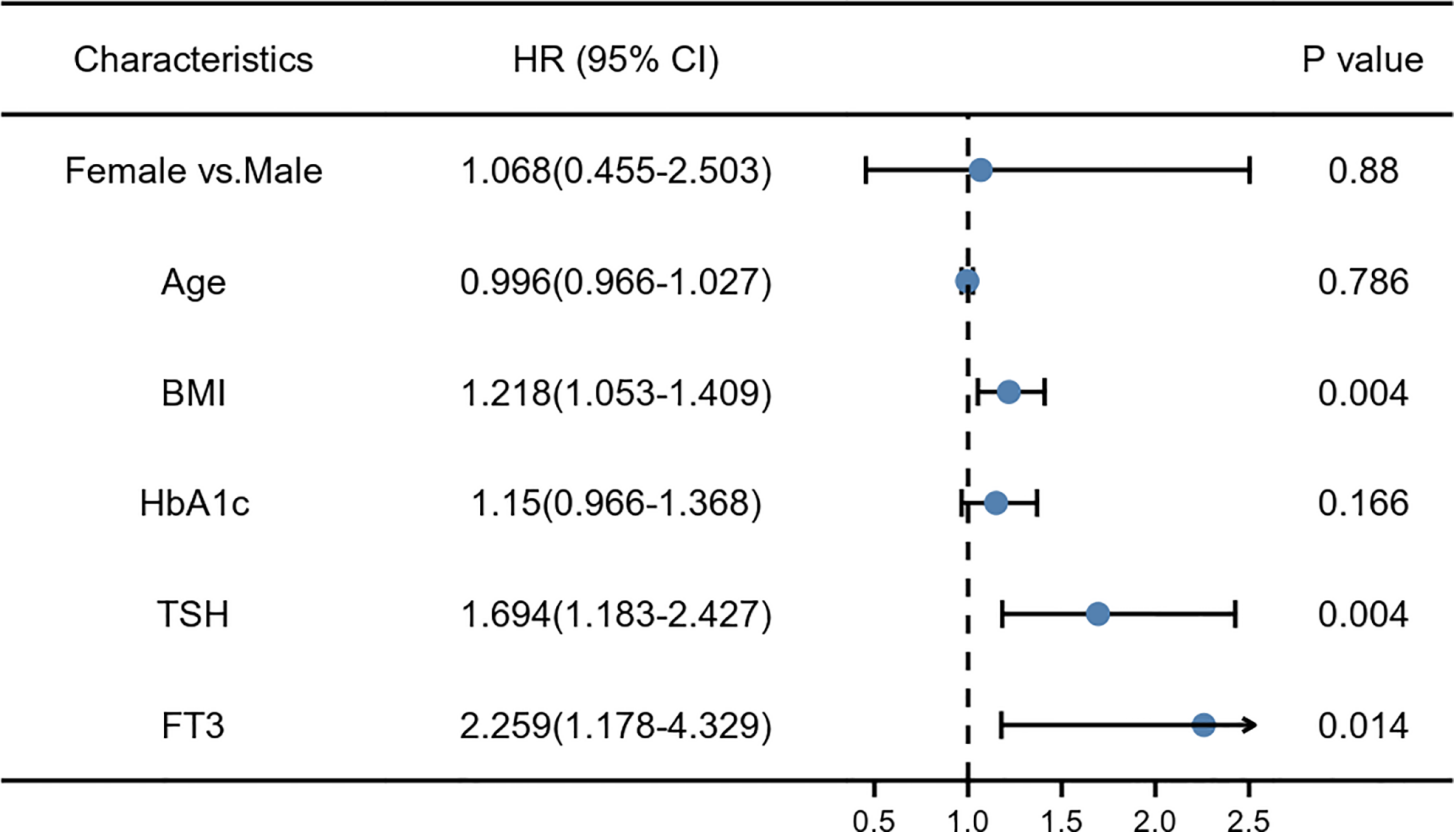
The multivariate logistic risk regression model: the risk factors for non-alcoholic fatty liver disease (NAFLD).

A multivariate logistic regression model was used to analyze the risk factors for NAFLD. After adjusting for all factors with a p-value less than 0.1 in the univariate analysis, BMI, TSH, and FT3 were identified as significant independent risk factors for NAFLD. The ORs were 1.218, 1.694, and 2.259, respectively (all p < 0.05) (**Figure 1**).

### Correlation and subgroup analysis

**Figure 2:**
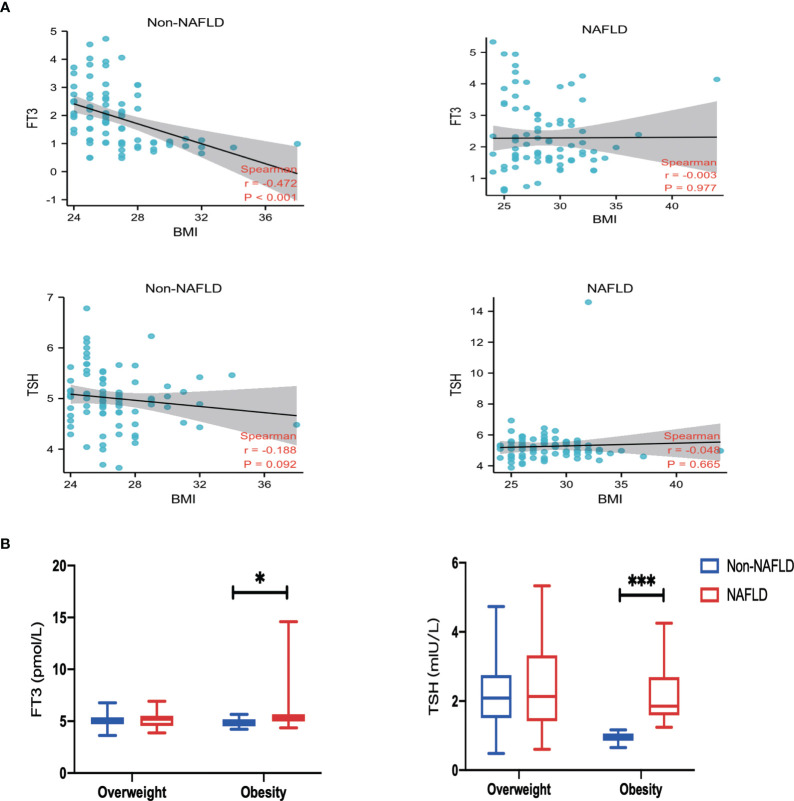
Correlation and Subgroup analysis. **(A)** Spearman correlation analysis of body mass index (BMI), free triiodothyronine (FT3) and thyroid-stimulating hormone (TSH). **(B)** Comparison of FT3 and TSH among groups classified according to BMI. Values are represented as mean ± SD. *p < 0.05, ***p < 0.001 vs. the Non-NAFLD group.

As FT3, TSH level, and BMI were correlated with NAFLD outcomes, the relationships between them were assessed. There was no significant relationship between BMI and TSH levels in NAFLD and Non-NAFLD group (r = −0.003 and -0.048, respectively). A weak negative correlation between BMI and FT3 was found in the NAFLD patients (r = −0.472, p < 0.001), as shown in **Figure 2A**. Further analysis with BMI stratification was conducted to compare the serum concentrations of FT3 and TSH in the two groups. Both FT3 and TSH levels were significantly different between individuals with and without NAFLD in the obesity subgroup; however, there was no significant difference in the overweight group (**Figure 2B**). Collectively, these findings indicate that the combination of NAFLD with obesity may have a TH resistance-like manifestation.

### Visualization of thyroid hormone signaling pathway in GSE48452

**Figure 3:**
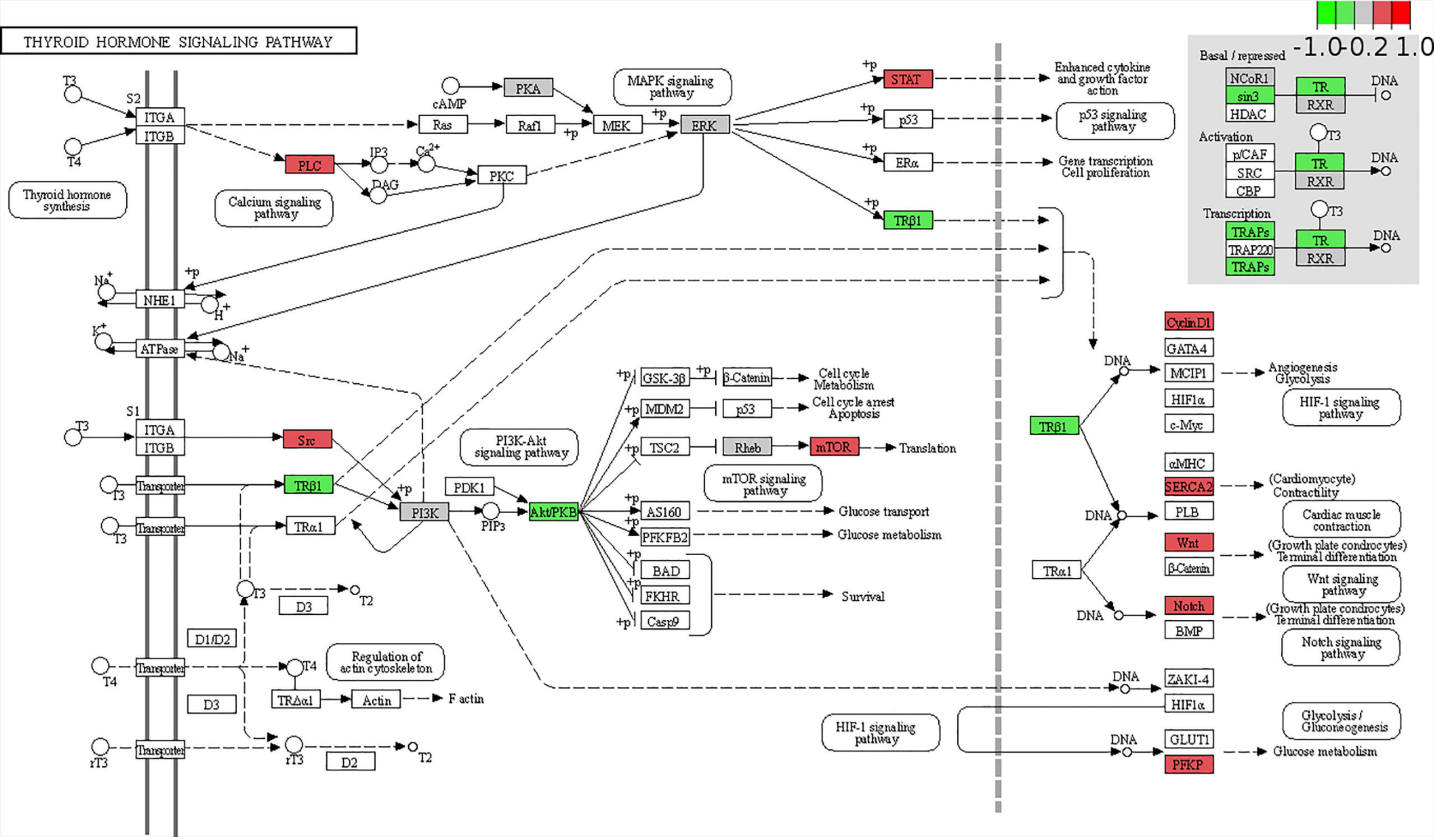
Expression changes of target genes of thyroid hormone signaling pathway in patients with non-alcoholic fatty liver disease (NAFLD) are mapped by colors. The color depth positively correlated with the degree value. Red represents increased while green represents decreased gene expression in NAFLD group compared with that in the health obese group.

Considering its important metabolic role, the liver may be the main cause of thyroid hormone resistance. To identify thyroid hormone signaling pathway changes in obese patients with NAFLD, we downloaded relevant expression profiles from the GSE48452 dataset. The Pathview library was used to visualize the thyroid hormone signaling pathway (18). **Figure 3** shows several key molecular pathways affected by NAFLD-induced transcriptomic changes in the liver samples from obese patients. The results show the expression of *THRB*, which encodes TRβ, was significantly decreased in the NAFLD group, which may explain the TH resistance-like manifestation in the clinical findings.

### Expression analysis of the DEGs and Immune cell infiltration in GSE48452

**Figure 4:**
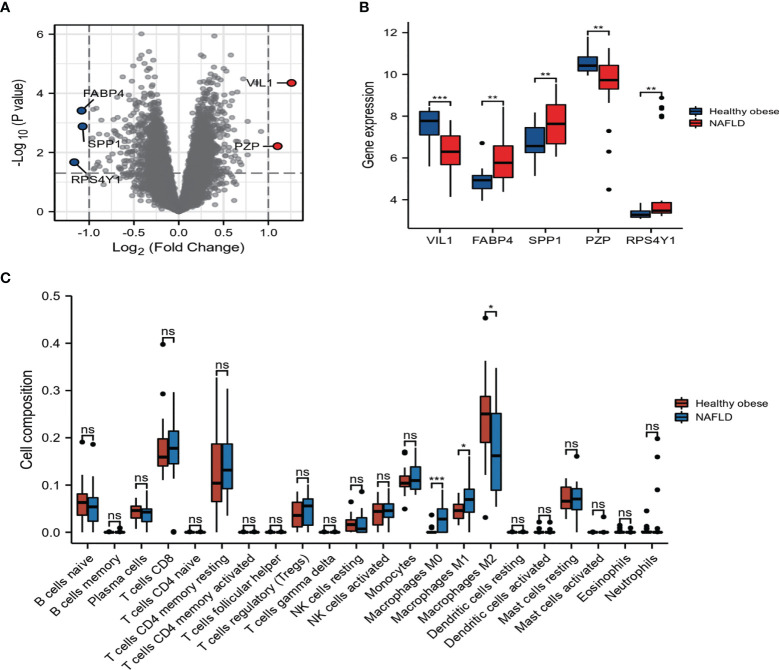
Expression and correlation analysis of the differentially expressed genes (DEGs). **(A)** DEGs in non-alcoholic fatty liver disease (NAFLD) and healthy obese samples in Gene Expression Omnibus (GEO) dataset. **(B)** The boxplot of five DEGs. **(C)** Bar plot showing the difference between 22 infiltrated immune cells in the NAFLD and healthy obese groups with the CIBERSORT algorithm. Values are the mean ± SD. ns, no significance, *p < 0.05, **p < 0.01, ***p < 0.001 vs. the healthy obese group.

To identify DEGs linked to NAFLD incidence, we downloaded relevant expression profiles from the GSE48452 dataset. Five DEGs involved in the development of NAFLD were identified by limma package (adjusted p < 0.05, |logFC| ≥ 1), as shown in the volcano plot (**Figure 4A**). Box plots show the expression patterns of the five DEGs between NAFLD and healthy obese individuals in the GSE48452 dataset (**Figure 4B**). The expression levels of *SPP1*, fatty acid binding protein 4 (*FABP4*), and ribosomal protein S4 Y-linked 1 (*RPS4Y1*) were significantly higher, whereas the expression levels of pregnancy zone protein (*PZP*) and villin 1 (*VIL1*) were significantly decreased in patients with NAFLD. Inflammation plays a vital role in the pathogenesis of NAFLD. Thus, understanding immune cell infiltration may provide a more comprehensive view of the efficacy of NAFLD therapies. The CIBERSORT algorithm showed increased M0 and M1, and decreased M2 macrophage infiltration in the NAFLD group compared to that in the healthy obese group (**Figure 4C**). These results indicate that abnormal macrophage polarization may play a vital role in fat deposition in hepatocytes.

### Validation of related clinical phenotypes and gene expression in NAFLD mice model

**Figure 5:**
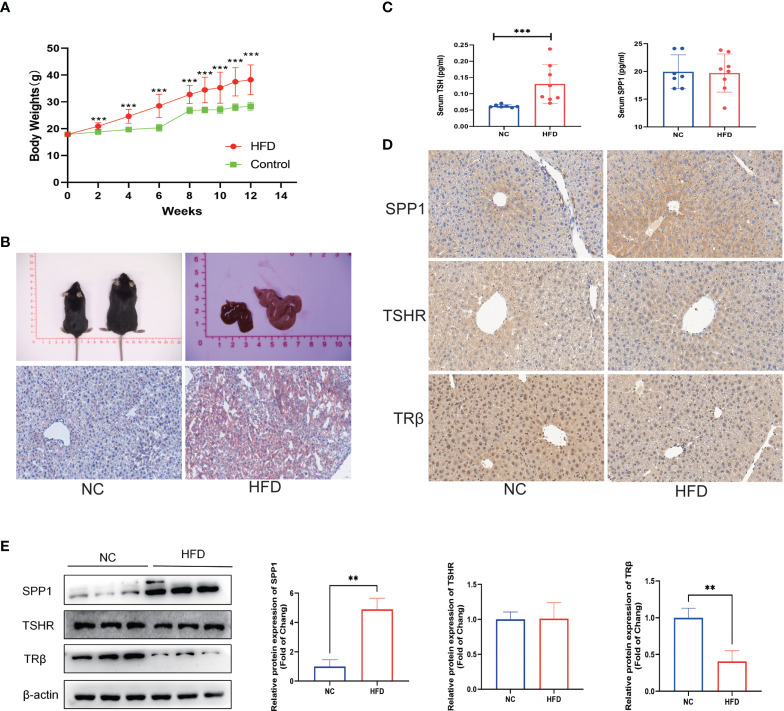
Validation of related clinical phenotypes and gene expressions in a non-alcoholic fatty liver disease (NAFLD) mice model. **(A)** Body weights of high-fat diet (HFD) and negative control (NC) mice over 12 weeks. **(B)** Representative images of the gross morphology of the liver and liver tissue sections stained with Oil Red O. **(C)** Enzyme-linked immunosprobent assay (ELISA) of serum secreted phosphoprotein 1 (SPP1) and thyroid-stimulating hormone (TSH). **(D)** Immunohistochemistry (IHC) staining of SPP1, TSH receptor (TSHR), and TRβ in mice livers. **(E)** Quantification of the average band densities calculated from western blots of SPP1, TSHR, and TRβ in the liver tissues from HFD and NC mice. Values are the mean ± SD. n = 8 mice in each the HFD and NC groups. **p < 0.01, ***p < 0.001 vs. NC.

An animal model of obesity was established by feeding male C57BL/6 mice with an HFD, and these mice were used to validate the related gene expressions and NAFLD phenotype found in the clinical data. As shown in **Figures 5A, B**, HFD feeding induced a significant increase in the body mass of the mice and exhibited a uniformly pale red fatty liver, while the NC group remained lean and had a relatively normal liver. Correspondingly, HFD feeding to mice showed increased fat accumulation in the hepatic intracellular vacuoles by Oil Red O staining. These mice had significantly higher serum TSH levels, which is in agreement with our clinical results, suggesting a TH resistance-like manifestation. However, there was no significant difference in the serum SPP1 concentration between the two groups (**Figure 5C**). To investigate SPP1, TSHR, and TRβ expressions in the liver, we analyzed IHC-stained liver sections (**Figure 5D**). The intensity of SPP1 immunostaining was significantly increased in HFD-fed mice compared to that in NC, while that of TRβ was reduced. The expression of TSHR did not significantly differ between the two groups. The expression levels of SPP1, TSHR, and TRβ were validated by western blotting (**Figure 5E**). Collectively, these findings further support that obesity was accompanied by NAFLD and TH resistance and that SPP1 and TRβ may be involved in this pathological process.

### SPP1 Induced TRβ downregulation and aggravated lipid accumulation in HepG2 Cells

**Figure 6:**
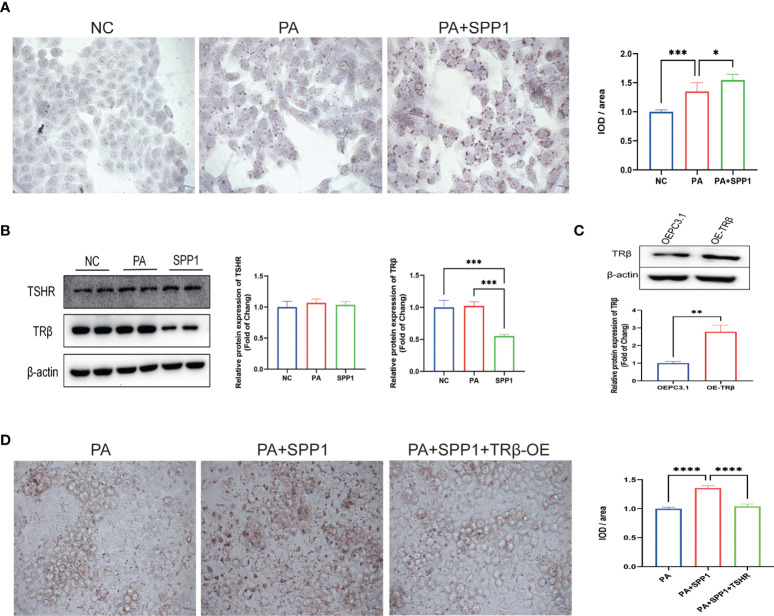
Secreted phosphoprotein 1 (SPP1) induced *THRB* downregulation aggravated lipid accumulation in HepG2 cells. **(A)** Lipid deposition in each group detected by Oil Red O staining. **(B)** Quantification of the average band densities calculated from western blots, the protein levels of TSH receptor (TSHR) and TRβ in these groups. **(C)** *THRB* was overexpressed in HepG2 cells and this was validated by western bloting. **(D)** Lipid deposition in those groups detected by Oil Red O staining. Values are the mean ± SD. n = 3 independent experiments. *p < 0.05, **p < 0.01, ***p < 0.001, ****p < 0.001.

To describe the relationship between SPP1, TRβ, and lipid deposition in hepatic cells, we examined HepG2 cells treated with 50 µM concentrations of PA and/or 20 ng/ml concentration of SPP1 for 48h. Oil Red O staining revealed a significant increase in lipid deposition in SPP1-stimulated cells as compared to that in the PA group (**Figure 6A**). In addition, the expression of TRβ, but not TSHR was significantly decreased after SPP1 administration (**Figure 6B**), suggesting that TRβ may be involved in SPP1-induced lipid deposition in the liver. To test this hypothesis, we modified TRβ levels in HepG2 cells by transfecting them with a plasmid overexpressing *THRB*, which was validated by western blotting (**Figure 6C**). Following *THRB* overexpression, there was a reversal in the phenotype of lipid accumulation following SPP1 treatment *in vitro* (**Figure 6D**). Collectively, these findings suggest that SPP1 aggravates lipid deposition in hepatic cells by downregulating *THRB* expression.

### TSH Acts to promote secretion of SPP1 in M1 macrophage cells

**Figure 7:**
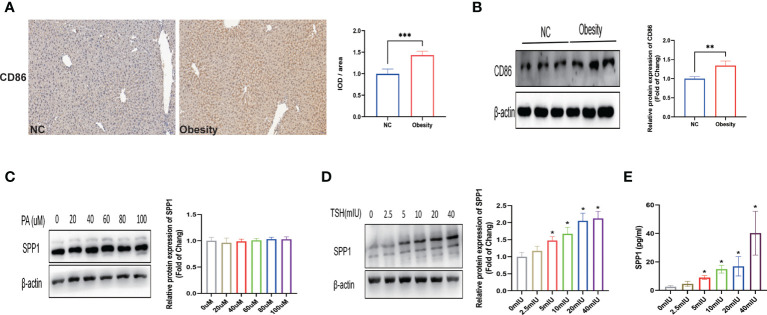
TSH acts to promote secretion of secreted phosphoprotein 1 (SPP1) in M1 macrophage cells. **(A)** Immunostaining of CD86 in mice livers. **(B)** Quantification of the average band densities calculated from western blots of CD86 in liver tissues. Protein levels of SPP1 stimulated with palmitic acid (PA) **(C)** or thyroid-stimulating hormone (TSH) **(D)** in M1 macrophage cells. **(E)** SPP1 in the supernatant after stimulation with TSH by ELISA. *p < 0.05, **p < 0.01, ***p < 0.001 vs. NC or 0 mIU TSH group.

Previous studies have shown that M1 macrophages play a key role in the process of metabolic inflammation underlying NAFLD development. To explore whether liver injury in obese mice is dependent on M1 macrophages, the distribution of M1 macrophages in the liver, indicated by the surface marker CD86, was evaluated by IHC and western blotting (**Figures 7A, B**). Both the intensity of immunostaining and expression of CD86 were significantly increased in the obese group, indicating that M1 macrophage polarization may be involved in intrahepatic inflammation. We used THP-1 cells to construct an M1 macrophage model *in vitro*. We then analyzed THP-1 cells treated with various concentrations of PA or TSH, and the assay revealed a significant dose-dependent increase in SPP1 expression or secretion in TSH-stimulated cells compared to that in control cells (p for trend < 0.05; p < 0.05 for 5 mIU or above; **Figures 7D, E**), while there were no significant changes with different PA administration (**Figure 7C**). Collectively, these findings suggest that increased TSH levels can further lead to the secretion of SPP1, thus maintaining and amplifying the pathological process of NAFLD.

## Discussion

In our clinical study, after adjusting for overweight/obese BMI and diabetes, the two most common causes of NAFLD, a manifestation of TH resistance, was found. This phenomenon was more obvious in obese patients, as shown in the subgroup analysis, suggesting that the intrahepatic damage of the TH pathway under obesity may play an important role in the occurrence of NAFLD. Bioinformatics analysis indicated that *THRB* was significantly downregulated in obese patients with NAFLD, and this phenotypic change was validated in an animal model with obesity. Further mechanistic studies demonstrated that HFD and PA induced increased secretion of SPP1 by M1 macrophages, which may downregulated TRβ in hepatocytes in a paracrine manner. This increased lipid deposition in the liver and a compensatory increase of TSH in serum. Increased TSH may lead to more secretion of SPP1, thus amplifying this pathological process. To the best of our knowledge, this is the first study to investigate the positive feedback crosstalk between the thyroid and the liver in NAFLD.

NAFLD is a serious worldwide health epidemic, which is causing a growing burden on public health (19). It is now well-established that NAFLD is accompanied by metabolic dysfunction, and increased BMI leading to obesity, and higher prevalence of obesity-related disorders, such as T2DM, in > 90% of patients (20). The liver and thyroid are intimately linked, with TH playing important roles in lipogenesis, beta-oxidation (fatty acid oxidation), and cholesterol metabolism (8, 21). Subclinical hypothyroidism and normal hypothyroidism have been found to be independent predictors of nonalcoholic steatohepatitis (NASH) (10, 22, 23). In this study, our multivariate analysis demonstrated that BMI, TSH, and FT3 were significant independent risk factors for NAFLD, and further analysis identified that BMI stratification showed that both FT3 and TSH had a significant change between individuals with NAFLD and the healthy liver obesity subgroup, indicating that NAFLD with obesity might have a thyroid hormone resistance-like manifestation. Chaves et al. provided evidence that impairments in liver TRβ signaling due to mutations in the *THRB* gene can lead to hepatic steatosis, which indicates the influence of TH on lipid metabolism in the liver (24). Similarly, TRβ impairment was also supported by subsequent bioinformatics and basic research in our study, which emphasize the important role of thyroid function in NAFLD in addition to the metabolic contributions of diabetes and obesity. Considering that the TH signaling pathway plays an important role in energy metabolism, researchers have been trying to pharmacologically target the FT3/THR axis for the past few decades. TH metabolites (25), TRβ agonists (26), and liver-specific analogs (27) have been studied as potential therapeutics for treating both serum dyslipidemia and as potential therapies for NAFLD. Resmetirom (MGL-3196) and Hep-Direct prodrug VK2809 (MB07811) probably representing two of the most promising lipid lowering agents, currently under phase 2-3 clinical trials. More recently the application of a comprehensive panel of ADME-Toxicity assays enabled the selection of novel thyromimetic IS25 and its prodrug TG68, as very powerful lipid lowering agents both (26, 28). Despite encouraging results in the treatment of obesity, dyslipidemia, and liver cancer, serious adverse reactions have limited their use in clinical trials. Determining the mechanism of TRβ damage will offer a better treatment option for NAFLD.

SPP1 is upregulated in obesity and several models of liver injury (29, 30).Studies have demonstrated a pivotal role of SPP1 in obesity-driven nutrition-dependent diseases, including NAFLD, suggesting SPP1 as a treatment target (13, 31). SPP1 animals showed enhanced hepatic lipid accumulation and aggravated NASH, as also increased hepatocellular apoptosis and accelerated fibrosis, which might be driven by enhanced hepatic fatty acid influx through CD36 overexpression. Lack of osteopontin lowered systemic inflammation, prevented HCC progression to less differentiated tumors and improved overall survival (13). In this study, we did not identify a statistically significant increase in serum SPP1 concentration after 12 weeks of HFD intervention, which may be because the challenge was too brief or too weak to cause a change. However, the HFD mouse model showed a significant increase in SPP1 expression in the liver when compared to that in the control group, which was consistent with the bioinformatics analysis in the GSE48452 dataset. SPP1 is an inflammatory cytokine highly upregulated in adipose tissue of obese and has repeatedly been shown to be functionally involved in adipose-tissue inflammation and metabolic sequelae (32). Although SPP1 promotes obesity and regulates lipid synthesis, both of which drive fat deposition in hepatocytes. However, further mechanistic studies are needed. Our findings show that downregulation of TRβ induced by SPP1 administration aggravated PA-induced lipid deposition in the liver, which was rescued by TRβ overexpression, indicating that SPP1-TRβ is involved in the pathological processes of NAFLD. TSH is produced by the hypothalamus and regulates thyroid hormone production (33). Studies have provide evidence that impairments in intrahepatic TRb signaling due to mutations of the THRB gene can lead to hepatic steatosis, which emphasizes the influence of TH in the liver metabolism of lipids (24, 34, 35). Likewise, our study showed a decrease in TRβ expression, but not TSHR, and a compensatory increase in TSH in both clinical patients and HFD mice with NAFLD. An interesting study demonstrated that liver steatosis and triglyceride content were significantly increased in TSHR^+/+^ HFD-fed mice compared to those in TSHR^−/−^ mice, indicating an essential role for TSH in the pathogenesis of NAFLD (36). However, this study did not involve hepatocyte-specific TSHR knockout. Combined with our current results, administration of different concentrations of TSH did not lead to SPP1 expression and lipid deposition in HepG2 cells, demonstrating that other cell types in the liver may be involved in this pathologic change.

While NAFLD can be driven by an imbalance between energy intake and expenditure (*e.g.*, diet and lack of exercise), inflammation is increasingly recognized as an important contributing factor (37). Such low-grade chronic inflammation induced by a high-fat diet or obesity propagates the pathogenesis of NAFLD-associated sequelae, comprising a spectrum from simple steatosis to NASH to end-stage cirrhosis and the risk of HCC, and has become a significant public health problem (38). Although certain extrahepatic sites, such as adipose tissue, have evolved as major sources of inflammatory mediators in obesity-related disorders, evidence is accumulating that intrahepatic inflammation might also be critically involved in the pathogenesis of NAFLD (39, 40). Although multiple cell populations in the liver contribute to various inflammatory pathways, hepatic macrophages are considered key contributors to the process of metabolic inflammation that underlies the development of NAFLD (41). The functional heterogeneity of monocyte-derived macrophages was initially typified by their polarization into M1 and M2 phenotypes, which were thought to represent two ends of a spectrum in which M1 macrophages are proinflammatory and M2 macrophages are regenerative (42). Weisberg et al. showed that the number of macrophages increased in the adipose tissue of mice and obese people and that this percentage was positively correlated with their obesity level (43). Macrophages isolated from white adipose tissue of lean animals showed characteristics of M2 macrophages (44). In addition, obesity-induced increased gene expression of characteristic molecules of M1 macrophages, such as those encoding TNF and NOS2, suggests that diet-induced obesity can lead to a shift in macrophage polarization from M2 to M1 (45). In our HFD-induced obese mouse model, similar results for M1 polarization were found, and further bioinformatics analysis showed that the differences in macrophage infiltration persisted even after correcting for obesity covariates, suggesting that M1 macrophage polarization may be involved in intrahepatic inflammation. Our results showed increased SPP1 expression and secretion as a consequence of M1 polarization, which may explain how M1 macrophages regulate TRβ expression in hepatocytes. Moreover, the increased TSH levels induced by TRβ damage can further lead to the secretion of SPP1, thus maintaining and amplifying this pathological process.

To the best of our knowledge, this is the first study to investigate the positive feedback crosstalk between the thyroid and the liver in NAFLD. The data presented in this study shed new light on NAFLD therapy. Our study had some limitations. First, there was a lack of liver-specific *TRHB* knockout or overexpression models to test the manifestation of thyroid hormone resistance in the NAFLD model. Second, we demonstrate a shift in macrophage polarization from M2 to M1 in NAFLD by bioinformatics analysis and in obese mice, but we did not explore the specific mechanism underlying this regulatory process. Hence, further investigations are needed to improve our understanding of the relationship between thyroid function and the liver, and whether M1 mediates SPP1-mediated lipid deposition in hepatocytes.

## Conclusions

This study establishes that SPP1 secretion is induced by M1 macrophage polarization, which may downregulates TRβ in hepatocytes in a paracrine manner. However, lipid deposition is aggravated in the liver, which causes a compensatory increase of TSH in serum. The increased TSH levels can further lead to SPP1 secretion by M1 macrophages. The positive feedback crosstalk between the thyroid and liver may play an important role in maintaining and amplifying the pathological process of NAFLD.

## Data availability statement

The raw data supporting the conclusions of this article will be made available by the authors, without undue reservation.

## Ethics statement

The studies involving human participants were reviewed and approved by the ethics committee of the First Affiliated Hospital of USTC, Division of Life Science and Medicine, University of Science and Technology of China. The patients/participants provided their written informed consent to participate in this study.

## Author contribution

HB performed the data acquisition and drafted the work. The main basic research was completed by WW. YS and HB interpreted the patient data. YS substantively revised it. All authors contributed to the article and approved the submitted version.

## Funding

This study was supported by the local scientific and technological development project guided by the central government of China (no. 2017070802D147)

## Conflict of interest

The authors declare that the research was conducted in the absence of any commercial or financial relationships that could be construed as a potential conflict of interest.

## Publisher’s note

All claims expressed in this article are solely those of the authors and do not necessarily represent those of their affiliated organizations, or those of the publisher, the editors and the reviewers. Any product that may be evaluated in this article, or claim that may be made by its manufacturer, is not guaranteed or endorsed by the publisher.
